# In this month’s Bulletin

**DOI:** 10.2471/BLT.22.000822

**Published:** 2022-08-01

In the editorial section Shannon Doocy et al. (466) call for a continuing crisis response in Venezuela. Claudia Chamas et al. (467) summarize recent initiatives to address diagnostic technology gaps in low- and middle-income countries.

Tatum Anderson (470–471) describes WHO’s work to improve reporting of attacks on health care in conflict settings. Ahmed Hankir talks to Vijay Shankar Balakrishnan (472–473) about his efforts to combat stigma surrounding mental health. 

## Brazil 

### Health workforce for the COVID-19 response

Ianka Cristina Celuppi et al. (520–524) introduce a database used to deploy health workers nationwide. 

## India 

### Addressing gestational diabetes 

Himanshu Nayak et al. (484–490) implement a screening programme in Ahmedabad.

## Islamic Republic of Iran 

### COVID-19 vaccine effectiveness

Ali Hosseinzadeh et al. (474–483) compare cases, hospitalizations and deaths.

## Saudi Arabia

### Ensuring access to medicines 

Khalid Almoteiry et al. (511–519) detail evolution and features of a national policy.

## Global

### Using vaccine thermostability to facilitate delivery

Christopher P Seaman et al. (491–502) review the evidence for the controlled temperature chain approach.

### Over-the-counter contraceptives 

Anne Ammerdorffer et al. (503–510) examine one way to improve access.

